# High-Concentration Time-Frequency Representation and Instantaneous Frequency Estimation of Frequency-Crossing Signals

**DOI:** 10.3390/s25072030

**Published:** 2025-03-24

**Authors:** Hui Li, Xiangxiang Zhu, Yingfei Wang, Xinpeng Cai, Zhuosheng Zhang

**Affiliations:** 1 School of Marine Science and Technology, Northwestern Polytechnical University, Xi’an 710072, China; lihui2018@nwpu.edu.cn; 2 School of Mathematics and Statistics, MOE Key Laboratory for Complexity Science in Aerospace, Northwestern Polytechnical University, Xi’an 710072, China; wangyf30198@mail.nwpu.edu.cn (Y.W.); caixinpeng@mail.npu.edu.cn (X.C.); 3 Shenzhen University of Advanced Technology, Shenzhen 518106, China

**Keywords:** time-frequency analysis, instantaneous frequency estimation, fully convolutional network, cross-over instantaneous frequency

## Abstract

Frequency-crossing signals are widely found in nature and various engineering systems. Currently, achieving high-resolution time-frequency (TF) representation and accurate instantaneous frequency (IF) estimation for these signals presents a challenge and is a significant area of research. This paper proposes a solution that includes a high-concentration TF representation network and an IF separation and estimation network, designed specifically for analyzing frequency-crossing signals using classical TF analysis and U-net techniques. Through TF data generation, the construction of a U-net, and training, the high-concentration TF representation network achieves high-resolution TF characterization of different frequency-crossing signals. The IF separation and estimation network, with its discriminant model, offers flexibility in determining the number of components within multi-component signals. Following this, the separation network model, with an equal number of components, is utilized for signal separation and IF estimation. Finally, a comparison is performed against the short-time Fourier transform, synchrosqueezing transform, and convolutional neural network. Experimental validation shows that our proposed approach achieves high TF concentration, exhibiting robust noise immunity and enabling precise characterization of the time-varying law of frequency-crossing signals.

## 1. Introduction

Widely present in fields such as sonar, radar, speech, biomedical science, and geophysics, multi-component non-stationary signals are often composed of different oscillatory components. Some of the component signals carry important information needed for practical applications. Therefore, it is necessary to accurately describe the instantaneous amplitude and frequency of each component signal of a multi-component non-stationary signal, which is conducive to gaining insights into the complex structure of the signal, understanding the system used to generate the signal, and predicting its future behavior. This is of much significance for various applications of the multi-component non-stationary signals [1,2,3,4].

In the analysis and processing of multi-component non-stationary signals, frequency-crossing signals—signals where IFs intersect in the TF domain—are commonly found in fields such as underwater acoustics, radar, and mechanical engineering. Examples of these include dolphin communication signals [5] and continuous-wave radar [6]. The critical challenge in addressing related application issues lies in effectively extracting the IF information from these frequency-crossing signals. TF analysis can be employed to track how frequency information varies over time, thereby aiding in the assessment of non-stationary signals [7,8]. However, the constraints of uncertainty principles and cross-interference terms lead to the low resolution of TF representation and the inaccurate characterization of signal features for various classical linear TF analysis methods such as short-time Fourier transform (STFT), wavelet transform (WT), and chirplet transform (CT) [9], as well as the quadratic TF analysis methods like Wiger Ville distribution (WVD) [7]. Consequently, achieving high accuracy in signal analysis becomes challenging.

In order to enhance the resolution of TF representation and improve the accuracy of information characterization, F. Auger [10] and I. Daubechies [11] proposed the TF rearrangement method and synchrosqueezing transforms (SSTs). Their methods not only achieve highly concentrated TF representations by rearranging or filtering the TF energy or coefficients of traditional TF transforms, such as STFT and WT, but also improve the accuracy of the TF location of non-stationary signals. However, this method can be considered as a post-processing transformation of the traditional methods of TF analysis. Its effect depends on the performance of traditional TF transformation, and it requires that multi-component signals meet certain separation conditions—specifically, that the IFs of each component signal are distinct and separable. Therefore, it is challenging to process the frequency-crossing signals, encountering such problems as the distortion of TF features and energy diffusion.

In recent years, there has been plenty of research conducted on the high-concentration TF representation and instantaneous frequency estimation of frequency-crossing signals. In reference [12], an iterative rearrangement algorithm was proposed to improve the readability of TF representations of intersecting regions. In references [13,14,15], CT was used to transform the signal to be analyzed into a three-dimensional space of time-frequency-chirp rate. This approach successfully separates frequency-crossing signals based on the differences in linear chirp rates (CR) between the components. Similarly, the IF estimation of frequency-crossing signals was achieved in references [5,16] by determining the CR values of the intersecting points of TF ridges. However, the TF basis functions used in these methods are fixed, which limits their effectiveness in feature representation. Currently, deep learning-based TF network methods have attracted widespread attention for obtaining upgradable TF basis functions. In reference [17], a convolutional autoencoder network was applied to enable the high-resolution TF representation of non-stationary signals. This network can automatically reduce the interference from cross-interference terms. For similar purposes, several WVD-based high-resolution TF network models were proposed to minimize cross-interference terms and achieve concentrated TF representation. These include a model that combines skipping 2D convolutional blocks with weighted blocks [18], a semi-supervised learning model based on the Mean Teacher approach [19], and a generative adversarial network [20]. In reference [21], an end-to-end network architecture called TFA net was proposed, with various TF characteristics of signals obtained by learning different basis functions. In reference [6], an AMTFN deep network was proposed to learn basis functions through multi-scale 1D convolutional kernels for the generation of TF feature maps. Then, communication channel attention mechanisms were incorporated into AMTFN to selectively rescale the TF feature maps.

Despite the significant progress made in the analysis and processing of frequency-crossing signals, there are still some outstanding problems. Firstly, current methods often produce inaccurate TF characterization when handling multi-component signals, particularly those with slight variations in CR values under noisy environments. It is still necessary to develop different TF networks that demonstrate strong generalization capabilities and robustess, fast processing time, and suitability for small sample sizes [22,23]. Secondly, the IF extraction algorithms based on TF representation lack adaptive ability. Therefore, it is necessary to set the parameters of the algorithm according to the specific features of the signal. Thirdly, the existing methods of neural network-based frequency-crossing signals analysis mainly focus on achieving high-resolution TF representations. However, they tend to overlook the development of models and networks applicable to extract the IFs of frequency-crossing signals.

To address the issues associated with frequency-crossing signals, a deep learning-based model for high-resolution TF feature characterization and IF estimation is developed. The main contributions of this paper are summarized as follows:

(1) A high-concentration TF representation model based on U-Net [24] is established to analyze frequency-crossing signals, expanding U-Net’s application in signal analysis. Unlike existing WVD-based and TF basis function learning models, the proposed model excludes the influence of cross-terms of WVD and performs feature fusion between low-resolution and high-resolution TF features to capture crucial TF details and reduce the information loss caused by an excessive focus on TF high-concentration.

(2) An IF separation and estimation network is constructed to determine the number of components in frequency-crossing signals and to adaptively extract the crossed IFs.

(3) The advantages of each design in the proposed model are validated through comparative analysis. Experimental studies demonstrate that the proposed approaches yield TF representations with high energy concentration and a noise-free background, successfully identifying the crossed IFs.

The rest of this paper is organized as follows: Section 2 provides a brief review of the frequency-crossing signal model and the STFT. Section 3 and Section 4 introduce the developed networks for high-concentration time-frequency representation and instantaneous frequency separation and estimation, respectively. In Section 5, we present the simulation results along with the processing results for a real bat echolocation signal. Finally, we conclude and discuss our findings in Section 6.

## 2. Signal Model and Short-Time Fourier Transform

In reality, signals are usually of multi-component, non-stationary nature, which means that the amplitude and frequency of the signals change constantly over time. In mathematics, they can be modeled as the sum of multiple modes of amplitude modulation and frequency modulation, for which they are known as multi-component non-stationary signals. For a *P*-component non-stationary signal, the mathematical model is expressed as follows:

(1) $$x\left(t\right)=\sum_{k=1}^{P}x_{k}\left(t\right)=\sum_{k=1}^{P}a_{k}\left(t\right)e^{j\phi_{k}\left(t\right)}$$

where $a_{k}\left(t\right)$ and $\phi_{k}\left(t\right)$ represent the instantaneous amplitude and instantaneous phase of the component signal $x_{k}\left(t\right)$, respectively, and *j* represents an imaginary unit. The instantaneous frequency of the *k*-th component is expressed as $\phi_{k}^{\prime }\left(t\right)/2\pi$.

If there are $t_{0}$, and *m*, *n*, m≠n, such that $\phi_{m}^{\prime }\left(t_{0}\right)=\phi_{n}^{\prime }\left(t_{0}\right)$, for which the signal is referred to as a frequency-crossing signal. Accurately characterizing and separating such signals presents a challenge in non-stationary signal analysis.

In this paper, TF data are created through a simple and intuitive short-time Fourier transform (STFT) without cross-term interference. By applying a window function w(τ), time shift and frequency modulation are performed to obtain the TF basis function $w\left(\tau -t\right)e^{j\tau f}$. Then, the mathematical definition of STFT is established as follows:

(2) $$S_{x}^{w}\left(t,f\right)=\int_{-\infty }^{+\infty }x\left(\tau \right)w*\left(\tau -t\right)e^{-j2\pi \tau f}d\tau$$

where the superscript ∗ denotes the complex conjugate. In this definition, the STFT is the process of extracting the local frequency spectrum of a signal x(t) through the window function w(τ−t), which reflects the relationship between the signal’s frequency spectrum and time.

## 3. High-Concentration TF Representation Network of Frequency-Crossing Signals

Fully convolutional networks (FCNs) [25] can be applied to solve image segmentation problems at the semantic level by classifying images at the pixel level. By replacing the fully connected layers of classic convolutional neural networks (CNNs) with convolutional layers, FCN can accept the input images of any size and output the feature maps of the corresponding size, rather than generating a fixed-length feature vector as was previously done. Through a series of operations such as convolution, downsampling, convolution, upsampling, and deconvolution, FCN can predict each pixel of an input image while maintaining the relative position of features of the original image. Therefore, this method requires fewer training images to complete image segmentation more accurately.

**Figure 1:**
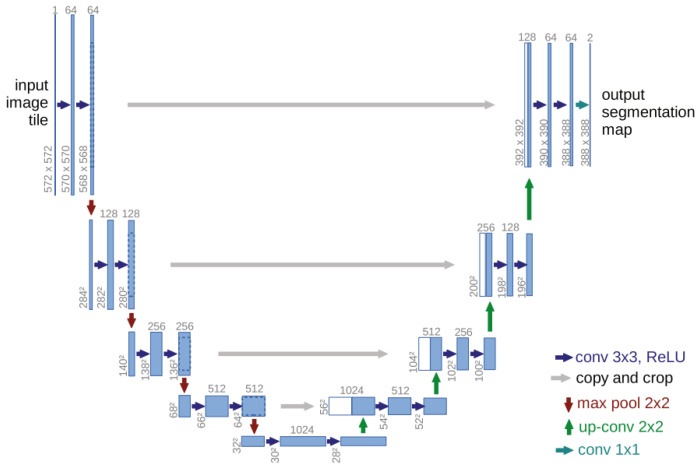
U-net structure [24].

Compared to FCNs, U-Net directly connects intermediate features from the encoder layers to corresponding layers in the decoder, preserving low-resolution details. This architecture enables superior accuracy, computational efficiency, and robustness to noise and low-contrast images [26]. Figure 1 shows the U-net structure.

**Figure 2:**
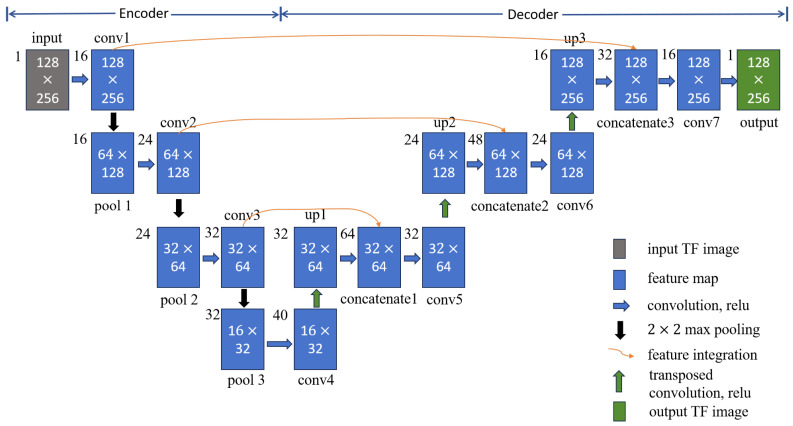
High-concentration TF representation network architecture.

Building upon the advantages of the U-net and its effective applications in medical image segmentation [26], WVD enhancement [27], and infrared small object detection [28], this section extends its design to achieve noise-robust and high-concentration TF representation of frequency-crossing signals. The structure of the developed network, named the high-concentration TF representation network, is shown in Figure 2, using a 128 × 256 TF map as a representative case. When the TF map of energy diffusion is inputted, feature maps are generated through convolutional and pooling layers. Dimensionality reduction is also applied to these feature maps to decrease computational complexity and reduce the risk of overfitting. Following this, transpose convolution and upsampling techniques are employed to restore the size of the feature maps progressively. The feature maps produced through pooling and upsampling, which are of the same size, are then concatenated along the depth dimension to avoid strong energy signal dominance and enhance the detailed features of the TF map. This process of upsampling, concatenation, and deconvolution is repeated until an output image is generated that matches the size of the original TF map.

Indeed, the high-concentration TF representation network is composed of an encoder layer and a decoder layer, as noted in Figure 2. In the encoding layer, there are three convolutional layers (conv1, conv2, conv3) and three pooling layers (pool1, pool2, pool3), with a convolution kernel size of 5 × 5. The decoding layer consists of three transposed convolutional layers (conv4, conv5, conv6) and upsampling layers (up1, up2, up3), which are purposed to extract detailed features. In addition, the network is applicable for performing feature fusion between the pooled feature map and the upsampled feature map (concatenate1, concatenate2, concatenate3), which further supplements the detailed features with a 3 × 3 convolution kernel. Finally, a 5 × 5 convolution kernel (conv7) is used to achieve high-concentration TF representation for the signal of the same size as the original TF map. Table 1 lists the number and size of convolution kernels in the high-concentration TF representation network.

**Figure 3:**
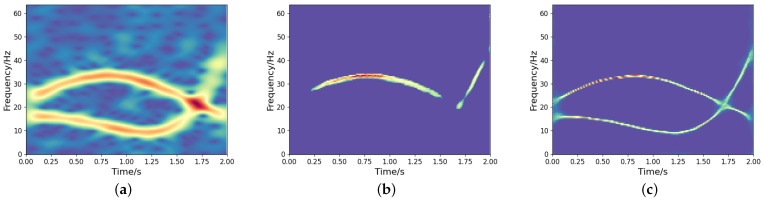
TF analysis of a noisy frequency-crossing signal. (**a**) The STFT of the signal; (**b**) TF output without concatenation; (**c**) TF output by the proposed model.

To investigate the impact of the feature fusion (i.e., concatenate1, concatenate2, concatenate3) on TF output, we conduct comparative experiments using two networks: the proposed high-resolution TF representation network and its counterpart without concatenation. A noisy frequency-crossing signal is processed through both architectures, and the STFT of the signal and its experimental results are presented in Figure 3. The results demonstrate that feature fusion effectively utilizes low-resolution TF features while suppressing the information loss associated with the one-sided pursuit of high-concentration of strong energy parts, resulting in an accurate and concentrated TF distribution. This highlights its crucial role in maintaining coherent TF representations under noisy conditions.

## 4. Instantaneous Frequency Separation and Estimation Network

Given the IFs of a signal, the instantaneous amplitude of the signal can be determined by solving the least squares problem:

(3) $$\begin{array}{c} \min_{a_{1},a_{2},\cdots ,a_{P}}{\left\|x-\left[\mathrm{diag}\left(e^{j\Phi_{1}}\right)\;\mathrm{diag}\left(e^{j\Phi_{2}}\right)\cdots \mathrm{diag}\left(e^{j\Phi_{P}}\right)\right]{\left[a_{1}\;a_{2}\cdots a_{P}\right]}^{T}\right\|}_{2}^{2} \end{array}$$

**Figure 4:**
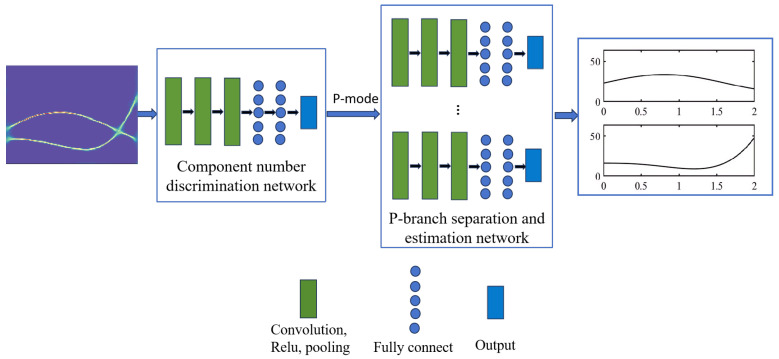
The architecture of the developed network for IF detection.

In this equation, x, $a_{p}$, and $\Phi_{p}$ denote the discrete signal, amplitude, and phase after sampling, respectively. The function diag(·) indicates the diagonalization of the vector, while ${\left[\cdot \right]}^{T}$ denotes the transposition. Therefore, accurately estimating the IF of a frequency-crossing signal is essential for analyzing non-stationary signals. However, unlike the research on high-resolution TF analysis of frequency-crossing signals, less attention has been paid to exploring the instantaneous information extraction methods for crossing signals. In light of this section, an IF separation and estimation network is proposed for frequency-crossing signals. Firstly, a discriminative network is constructed to determine the number of signal components. On this basis, an IF separation and estimation network suitable for frequency-crossing signals is built to extract the IF of each component of a signal. The architecture of the developed network is illustrated in Figure 4.

### 4.1. Determination of Number of Components

**Figure 5:**
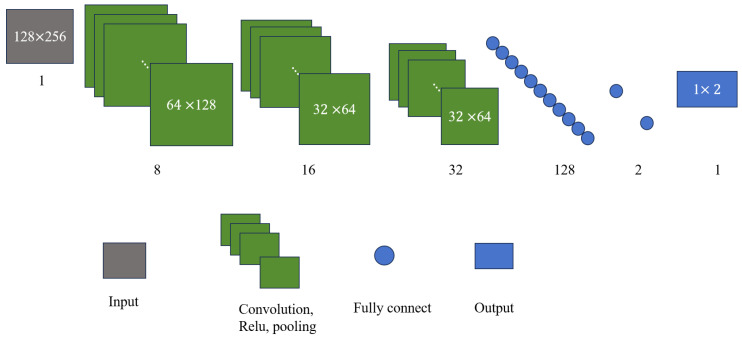
Component number discrimination network for two-component signals.

In this section, a CNN model is established to determine the number of components in a multi-component frequency-crossing signal and to confirm the number of branches to be used in the separation model. Assume that the number of components in a signal is 2 or 3 and that the input of the model is a 128 × 256 high-concentration TF image. Building on this, we construct the component number discrimination network, as illustrated in Figure 5. The network consists of three convolutional layers, each followed by pooling layers, using a 3 × 3 convolution kernel. After a fully connected layer is passed through, the features are mapped into a vector that represents the probability value of the number of components.

### 4.2. Extraction of Instantaneous Frequency

**Figure 6:**
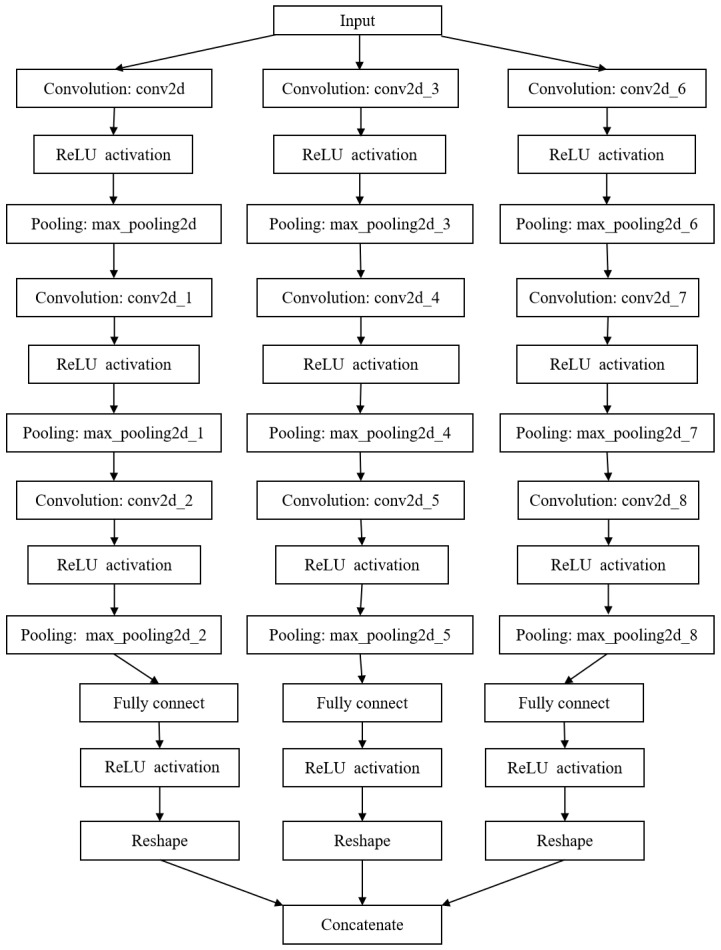
Instantaneous frequency separation and estimation network for three-component signals.

As described in the previous section, the number of components in a multi-component signal can be determined with the component number discrimination network, which is set as P. A P-branch separation network is constructed to separate the IF information of each component signal from the high-concentration TF representation. Each branch consists of a convolutional layer, a ReLU activation layer, and a max pooling layer. The TF features are mapped into a vector of the same length as the signal passes through the fully connected layer. Finally, the vector is concatenated into an IF estimation matrix, as illustrated in Figure 4. Figure 6 shows the basic architecture of the IF separation and estimation network of a frequency-crossing signal with three components. This network involves three single-branch models. Table 2 lists the parameter settings of the model’s convolutional layer.

## 5. Network Training and Numerical Results

### 5.1. Data Generation

**Figure 7:**
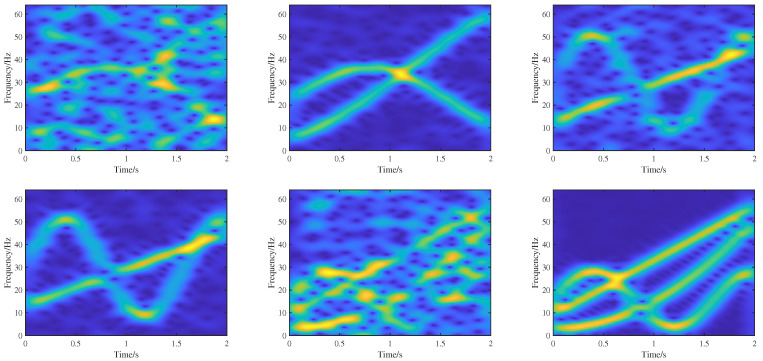
Six TF images featuring different models and noise levels.

This paper focuses on two-component and three-component signals using a mathematical model that is expressed as follows:

(4) $$\begin{array}{cc} \; & x\left(t\right)=\sum_{k=1}^{n}x_{k}\left(t\right)+g\left(t\right),\;n=2\;\mathrm{or}\;n=3\\ \; & x_{1}\left(t\right),x_{2}\left(t\right)=\cos\left(2\pi \left(a+bt+ct^{2}+dt^{3}+et^{4}+ht^{5}\right)\right)\\ \; & x_{3}\left(t\right)=\sin\left(2\pi \left(\frac{a}{\pi }\cos\left(b\pi t+\pi \right)+ct\right)\right) \end{array}$$

where g(t) is the Gaussian noise, the signal sampling frequency is set as 128 Hz, with a sampling duration of 2 seconds, a,b,c,d,e,h are signal parameters randomly generated from [−20 50] making the true IF values between 0 and a half of the sampling frequency. For each group of signals, there are 10 different signal-to-noise ratios (SNRs) of noise signals, including −10 dB −5 dB, 1 dB, 5 dB, 10 dB, 15 dB, 20 dB, 25 dB, 30 dB, and 35 dB. The total number of sample signals is 10,000. The STFT with a Hanning window (57 points) is performed on these signals to generate 10,000 TF images with a size of 128 × 256. With 9000 images taken as the training set, the remaining 1000 images are taken as the validation set. During the training process, we utilize ideal TF representations as labeled data for the high-concentration TF representation network, while real IF information served as labeled data for the IF separation and estimation network. Figure 7 shows six TF training samples.

### 5.2. High-Concentration TF Representation Results

**Figure 8:**
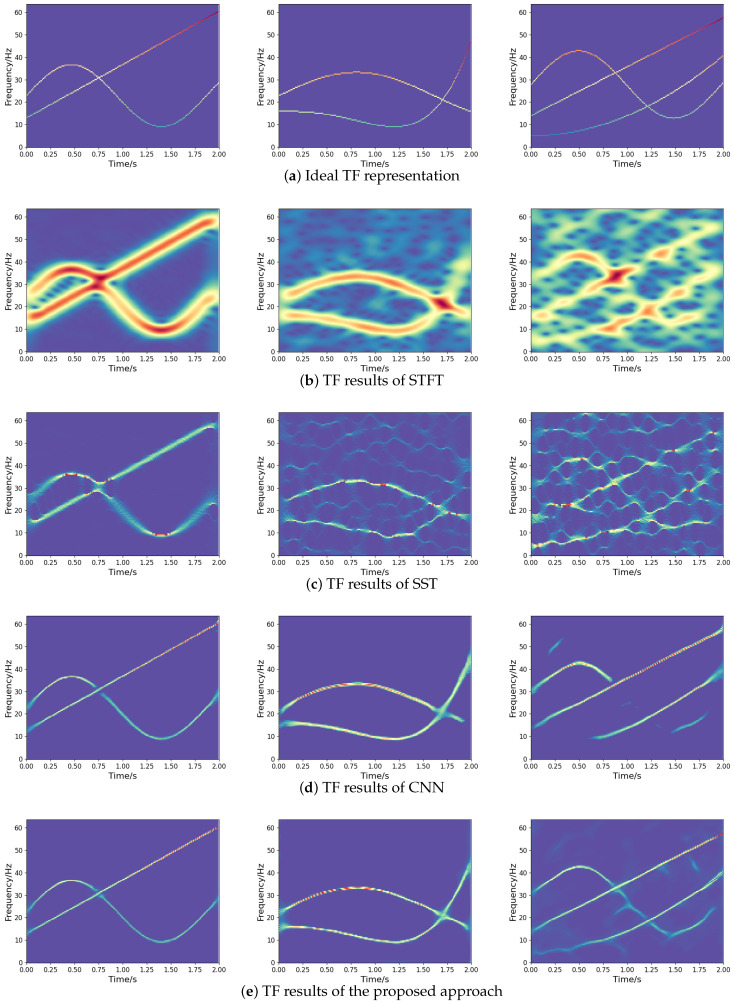
TF representations of different methods for noisy frequency-crossing signals.

The loss function used in this section is the mean square loss (MSE) function, of which its formula is expressed as follows:

(5) $$\mathrm{loss}\left(x,y\right)=\frac{\sum_{i=1}^{N}{\left(x_{i}-y_{i}\right)}^{2}}{N}$$

where $x=[x_{1},x_{2},\cdots ,x_{N}]$ denote the output, and $y=[y_{1},y_{2},\cdots ,y_{N}]$ denote the ideal TF representation, as presented in Figure 8a. The optimizer relies on Adam for optimization, with an initial learning rate of 0.001. During the training phase, the dataset is divided into batches of 32 and trained 500 times. In order to better demonstrate the effectiveness of the network, the experimental results are compared with STFT, synchrosqueezing transform (SST) [11], and TF network based on CNN [17] (abbreviated as CNN). The experimental results are presented in Figure 8.

As revealed by the numerical experiments, the STFT method leads to a lower TF resolution for frequency-crossing signals, while the SST method improves the TF resolution of STFT. In spite of this, some problems remain, such as energy diffusion and feature blurring. Obtained by inputting the TF dataset generated in this paper for training, the experimental results of CNN are not as expected, due to energy diffusion and information loss. Compared to other methods, the method proposed in this paper is more effective in TF representation, which improves the TF concentration greatly and enables a better characterization for noisy frequency-crossing signals. This contributes an effective approach to the information representation of multi-component non-stationary signals.

For the quantitative evaluation of TF analysis methods, we assess the time cost, mean absolute error (MAE), and Rényi entropy. The smaller the MAE, the closer the output is to the ideal one, and a lower Rényi entropy value indicates a more energy-concentrated TF representation, as discussed in [22]. The results are summarized in Table 3, where we apply these methods to the second two-component signal shown in Figure 8. The findings illustrate that TF networks, such as CNN and our proposed approach, increase the computational complexity of the traditional method of TF analysis. However, the proposed approach requires less time to represent the signal than CNN. Moreover, the proposed approach has lower MAE and Rényi entropy, which indicates better TF location and energy concentration than STFT, SST, and CNN.

To test the noise robustness and characterization accuracy of our proposed approach, we use the MSE to compare the performance of the analysis methods under different SNRs. The test signal of the following model is processed:

(6) $$x\left(t\right)=5\exp\left(-0.002t^{2}\right)\sin\left(2\pi \left(25t-5t^{2}+4t^{3}\right)\right)+\left(6-0.5t\right)\sin\left(2\pi \left(50t+5t^{2}-4.5t^{3}\right)\right)+g\left(t\right)$$

**Figure 9:**
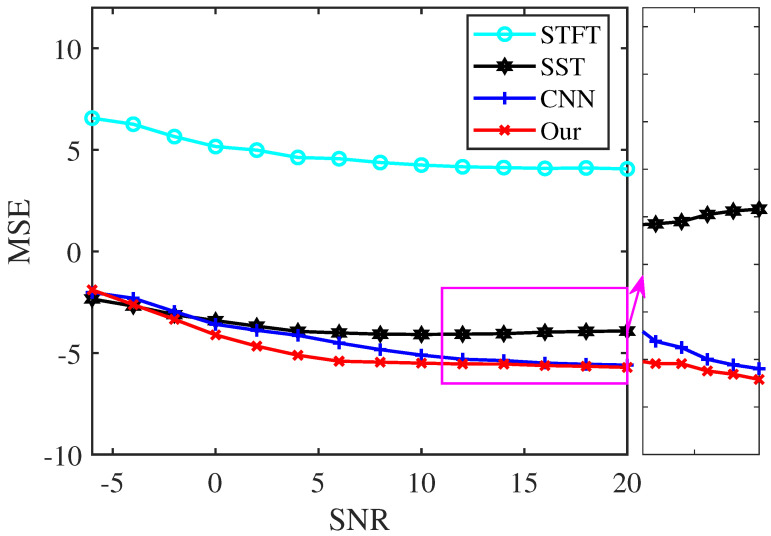
MSE of the TF results of signal (6) under different noise interference.

The sampling frequency is 256 Hz, and the signal is over a time interval of [0, 2 s]. Figure 9 displays the logarithmic MSEs between the TF representations by the four analysis methods and the ideal one, applied to signal (6) at various noise levels. The results demonstrate that our proposed method achieves smaller MSEs than STFT, SST, and CNN within the SNR range of −4 dB to 20 dB, indicating a more accurate TF location in characterizing the signal.

### 5.3. Instantaneous Frequency Extraction Results

**Figure 10:**
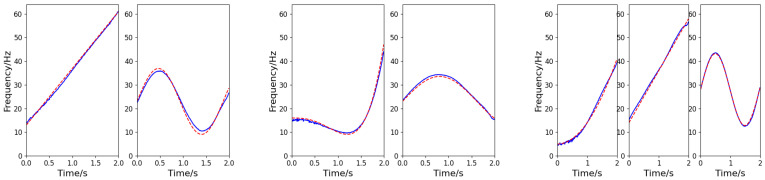
IF estimation of the frequency-crossing signals (the blue solid line represents the estimated value, and the red dashed line represents the actual value).

The component number discrimination network adopts the cross-entropy loss function for Adam optimization. The initial learning rate is 0.001, the batch size is 32, and there are 500 rounds of training. Based on the number of components identified by the discrimination network, a multi-branch instantaneous frequency separation and estimation network is established. The network adopts the MSE function for Adam optimization. The initial learning rate is 0.001, the training batch size is 32, and there are 300 rounds of training. The three signals exemplified in Section 5.2 are taken as example (SNR = −5 dB, 5 dB, and 10 dB for the first, second, and third signals, respectively). The output of the frequency-cross signal separation network is shown in Figure 10, in which each column corresponds to the IF information of each signal. According to the results of simulation experiment, the method developed in this paper can be used to effectively determine the number of components of multi-component cross signals and successfully extract the IFs of frequency-crossing signals, even in low SNR environment.

### 5.4. Practical Application

To examine the effectiveness of the proposed method in handling real-world signals, we consider the open-access bat echolocation data recorded by Rice University. This signal contains 400 samples with a sampling period of 7 μs.

**Figure 11:**
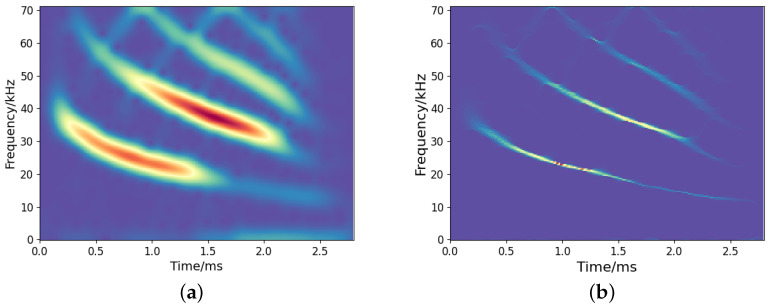

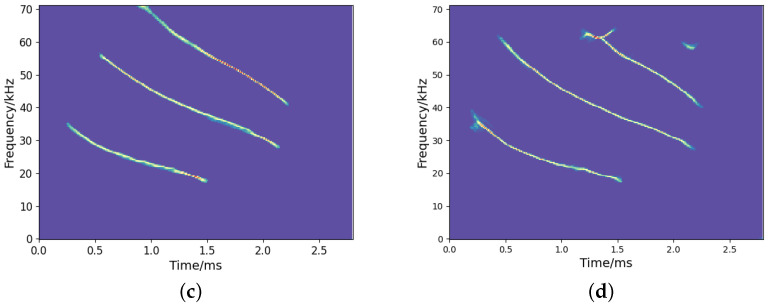
Results of TF representation of bat signals: (**a**) STFT; (**b**) SST; (**c**) CNN; (**d**) Our.

Figure 11 depicts the TF representations by the employed TF analysis methods. As presented in Figure 11a, the result of STFT is diffusive due to the limitation of uncertainty principles. SST yields a more sharpened TF representation than STFT, but there is still energy diffusion. Figure 11c,d show the TF representation by the CNN and our proposed method, which reflects a clear signal energy distribution with a high resolution. In comparison, our approach reveals more refined and detailed TF information.

## 6. Conclusions and Discussion

In this paper, a new high-concentration TF representation network and an instantaneous frequency separation and estimation network are proposed for analyzing and processing frequency-crossing signals. Under the effective feature extraction mode of U-net, the TF representation network can concentrate information on spectrograms of any size and achieve high-resolution TF representations of frequency-crossing signals. By implementing the component discrimination network alongside the instantaneous frequency separation and estimation network, the instantaneous frequency curves of each component signal of the frequency-crossing signal are effectively extracted. The experimental results show that the proposed method enables not only a more concentrated TF representation than STFT, SST, and CNN methods but also an accurate estimation of the time-varying law of frequency-crossing signals, highlighting the effectiveness of our approach.

**Figure 12:**
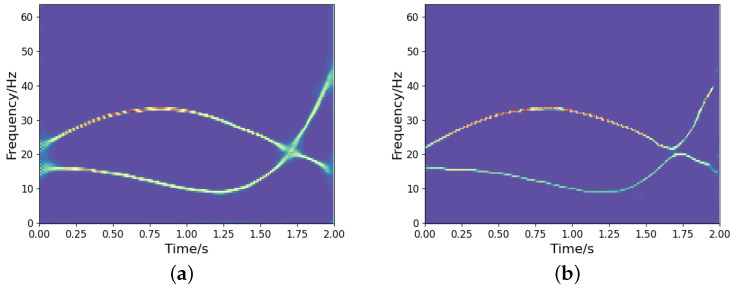
The high-concentration TF representation network over a deeper network for the noisy frequency-crossing signal in Figure 3. (**a**) TF output by the proposed model; (**b**) TF output by the model in [27].

**Figure 13:**
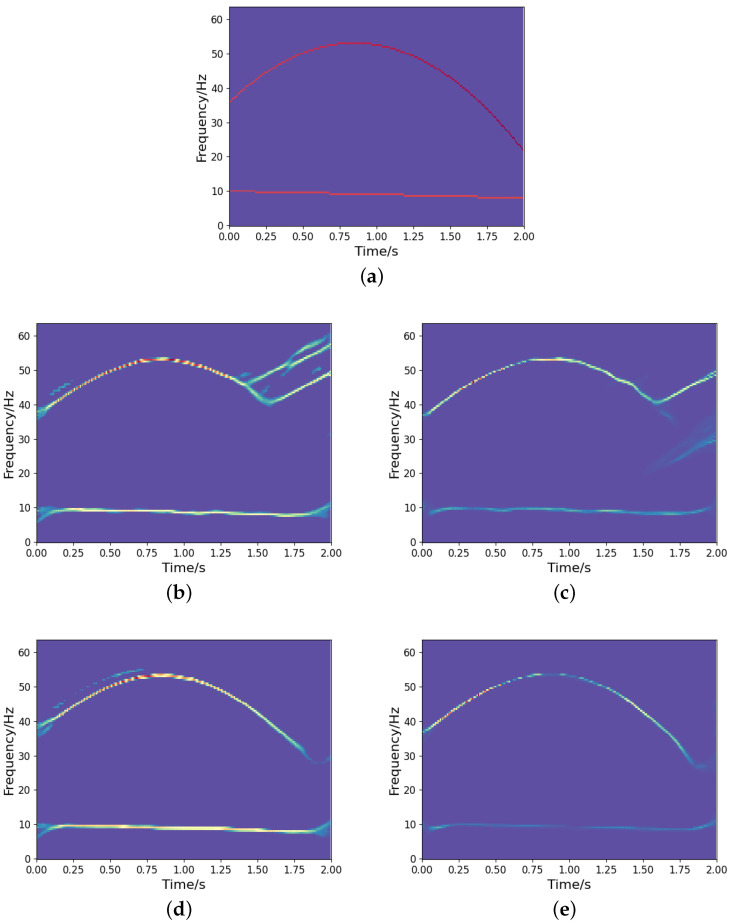
The results of TF representation of a frequency-uncrossed signal: (**a**) Ideal TF representation; (**b**) CNN output under SNR =−5 dB; (**c**) Output from the developed approach under SNR =−5 dB; (**d**) CNN output under SNR =15 dB; (**e**) Output from the developed approach under SNR =15 dB.

It is noted that we could implement a deeper network structure based on the proposed model, as outlined in [27]. A deeper network tends to yield a more concentrated TF representation. However, it may also result in the loss of certain signal information. Figure 12 shows the TF output by the deeper network model in [27]. We can observe that some signal information is distorted and missing compared to the ideal representation (see Figure 8a), even though it provides a more concentrated TF representation. Therefore, designing a learning network that can optimize the TF distribution of signals while fully characterizing the signal information is a crucial issue that deserves further investigation. In addition, to assess the generalization capability of the network, we test both the proposed high-resolution TF network and the CNN using frequency-uncrossed signals (its ideal TF representation is shown in Figure 13a). The experimental results are presented in Figure 13b–e. These outcomes indicate that our proposed model produces less interference than CNN; however, both networks show representation bias. Improving the generalization capability of networks remains a significant challenge for researchers. Moreover, similar to CNN and other techniques, the proposed method encounters substantial errors in estimating the IFs of frequency-crossing signals when strong noise is present. Therefore, further research is necessary to address this challenge.

## Figures and Tables

**Table 1:** Convolution layer parameters in the high-concentration TF representation network.

Layer	conv1	conv2	conv3	conv4	up1	concatenate1	conv5
Number	16	24	32	40	32	32	32
Size	5 × 5	5 × 5	5 × 5	5 × 5	5 × 5	3×3	5 × 5
**Layer**	**up2**	**concatenate2 **	**conv6**	**up3**	**concatenate3 **	**conv7**	
Number	24	24	24	16	16	16	
Size	5 × 5	3 × 3	5 × 5	5 × 5	3 × 3	5 × 5	

**Table 2:** Convolution layer parameters in the IF separation and estimation network.

Layer	conv2d, conv2d_3, conv2d_6	conv2d_1, conv2d_4, conv2d_7	conv2d_3, conv2d_5, conv2d_8
Number	8	10	16
Size	5 × 5	5 × 5	5 × 5

**Table 3:** Time cost, MAE, and Rényi entropy of the four TF representations in addressing the signal, where ·/step indicates the average processing time of each batch.

Method	STFT	SST	CNN	Our
Time cost (ms)	1.9	4.6	413/step	361/step
MAE	0.959	0.617	0.409	0.372
Rényi entropy	13.5	11.4	10.4	10.1

## Data Availability

The original contributions presented in this study are included in the article. Further inquiries can be directed to the corresponding author.
